# An Overview of Nucleic Acid Testing for the Novel Coronavirus SARS-CoV-2

**DOI:** 10.3389/fmed.2020.571709

**Published:** 2021-01-18

**Authors:** Huiling Wang, Gang Li, Jing Zhao, Yanjie Li, Yushu Ai

**Affiliations:** ^1^Department of Clinical Laboratory, Henan Provincial People's Hospital, People's Hospital of Zhengzhou University, People's Hospital of Henan University, Zhengzhou, China; ^2^Department of Research Management, Henan Provincial People's Hospital, People's Hospital of Zhengzhou University, People's Hospital of Henan University, Zhengzhou, China

**Keywords:** COVID-19, nucleic acid testing, SARS-CoV-2, qRT-PCR, specimen

## Abstract

In this note we analyze the problems in the nucleic acid testing (NAT) of severe acute respiratory syndrome coronavirus 2 (SARS-CoV-2), and we also give some suggestions for improving the accuracy of NAT diagnosis. NAT testing is considered to be the diagnostic “gold standard”; at present there are few reviews on NAT for SARS-CoV-2. Moreover, many false-negative results always appear in the procedure of detecting, which has affected early diagnosis of the disease and brought a great challenge to mitigation and containment of the pandemic. In conclusion, comprehensive analyses of serological and imaging findings should be performed to guide the formulation of an accurate clinical diagnosis, treatment plan, and monitoring therapeutic efficacy, in an effort to achieve early diagnosis, containment, and treatment of the disease, thereby effectively reducing progression of the pandemic. This article presents a literature overview of SARS-CoV-2 nucleic acid testing, aiming to provide support for clinicians.

## Introduction

The novel coronavirus pneumonia (COVID-19) pathogen is a severe acute respiratory syndrome coronavirus 2 (SARS-CoV-2). It is the seventh coronavirus identified in recent years that can infect humans, identified after the severe acute respiratory syndrome coronavirus (SARS-CoV) and Middle East respiratory syndrome coronavirus (MERS-CoV) (1). SARS-CoV-2 is a positive-sense single-stranded RNA virus that is highly contagious, with the general population lacking immunity to it (2). The COVID-19 pandemic is currently ongoing; on January 30, 2020 (local time), WHO declared this pandemic a Public Health Emergency of International Concern (PHEIC). This article presents a literature overview of COVID-19 nucleic acid testing, aiming to provide support for clinicians.

## Significance of SARS-CoV-2 Nucleic Acid Testing

Commonly used clinical methods used to test for viral pathogens include virus isolation and viral nucleic acid testing (NAT). Virus isolation is the “gold standard” for laboratory diagnosis, but it is far from meeting the clinical needs required for a large number of suspected patients in a short period of time. Moreover, virus isolation can only be performed in biosafety level 3 (BSL-3) laboratories or higher; conventional laboratories do not meet these requirements, and thereby it poses a great challenge in confirming a diagnosis. Procedures of NAT tests for RNA virus include RNA extraction, nucleic acid amplification, and target gene detection. Either polymerase chain reaction (PCR) or isothermal amplification can be used for nucleic acid amplification. Isothermal can be divided into many types, such as LAMP (loop-mediated isothermal amplification) and RPA (recombinase polymerase amplification). RPA is used in SHERLOCK system (CRISPR-cas13) (3), while LAMP is used for amplification in DETECTR assay (CRISPR-cas12) (4). A specific sequence(s) of the SARS-CoV-2 genome is amplified and detected with fluorescently labeled probe(s) by using the technique of quantitative real-time reverse transcription PCR (qRT-PCR), during which the patients' viral loads were detected. Detection by qRT-PCR is the most sensitive, specific, and simple diagnostic tool currently available. As confirmed in the first six versions of *the Diagnosis and Treatment Protocol for Novel Coronavirus Pneumonia*, released by the National Health Commission of China, a COVID-19 diagnosis can be confirmed using two approaches: (1) assessing positive NAT results by fluorescent quantitative real-time reverse transcription PCR (qRT-PCR) and (2) genome sequencing assessing high homology to SARS-CoV-2. Clinically, qRT-PCR is used to confirm diagnosis in the majority of suspected cases (5). Compared with gene sequencing (6), qRT-PCR is faster, can be used on a larger-scale, and is more affordable. As the pandemic progresses, a variety of SARS-CoV-2 NAT kits have been rapidly developed in China, most of which are based on qRT-PCR technology.

## Genome of SARS-CoV-2

**Figure 1:**
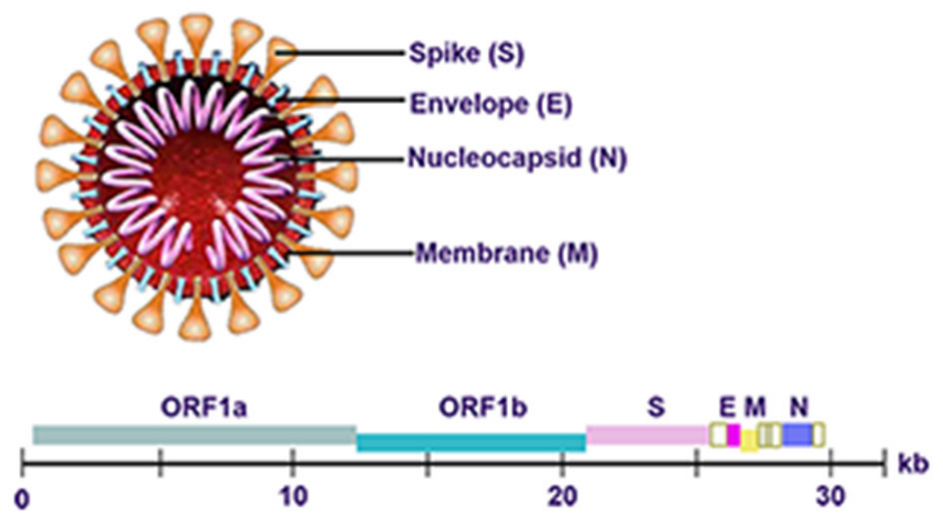
Genome of SARS-CoV-2.

The genome of SARS-CoV-2 is approximately 30 kb in length and consists of six open reading frames (ORFs), which includes ORF1a/b, spanning 16 non-structural proteins (nsp) relating to the replication-transcription complex, four structural proteins, spike (S), envelope (E), membrane (M), and nucleocapsid (N), along with several other non-structural, special structural, and/or accessory ORFs (ORF3a/b, 6, 7a, 7b, 8, and 10) (7–9) (Figure 1). Most diagnostic tests target a combination of structural (S, N, and/ or E) and non-structural (ORF1ab region) SARS-CoV-2 genes, along with positive and negative controls. This testing strategy ensures that the diagnostic targets include a nonstructural protein, highly conserved for coronaviruses, as well as structural protein(s), highly specific for SARS-CoV-2. WHO-recommended PCR assays can be designed to detect the sequence information from the SARS-CoV-2. Amplification and detection of specific sequences of SARS-CoV-2 can be diagnostic without the necessity for further sequencing (10). Different countries always have different recommendations about detecting genes for SARS-CoV-2 (Table 1). In summary, Chinese CDC recommended primers and probes targeting ORF1ab and N gene (11). In Germany, Charité recommended E gene assay as first-line screening assay with technical limit of detection (LOD) of 5.2 copies/reaction, and RdRp gene assay as confirmatory assay with technical LOD of 3.8 copies/reaction (5). The Ministry of Public Health of Thailand recommended to detect *N* gene (12). Japan recommended to detect ORF1a and S gene, as well as N gene (13). The US CDC has developed assays including three pairs of N gene of SARS-CoV-2 in early stage (14).

**Table 1:** Recommendations about the detecting genes for SARS-CoV-2.

**Institute**	**Gene targets**
Chinese CDC	ORF1ab, N
Germany Charité	E, RdRp
Ministry of Public Health of Thailand	N
Japan	ORF1a, S, N
US CDC	Three pairs of N gene

## Existing Problems of NAT for SARS-CoV-2

Despite that qRT-PCR testing had many advantages listed above, its results are subject to many influencing factors. Since the COVID-19 outbreak, NAT testing, which is considered the diagnostic “gold standard,” sometimes generates false-negative results due to many factors, which has affected early diagnosis of the disease and brought a great challenge to mitigation and containment of the pandemic. The major limitations of SARS-CoV-2 NAT are discussed below.

### NAT Kits Are Technically New

Upon the emergence of a new virus, a certain time period is required to develop a validated kit through isolation, sequencing, and identification. As knowledge of the disease increases, kits gradually become optimized, resulting in improved specificity and accuracy. The detection sensitivity of most qRT-PCR kits is between 100 and 500 viral copies in a reaction. As such, kits can only be used to detect high viral loads, and detection results may significantly be inconsistent across different manufactured kits or even among different batches of kits from the same manufacturer. Due to the need to contain the pandemic, SARS-CoV-2 NAT kits can directly be used to diagnosis COVID-19 before they are validated through a large amount of clinical trials, and the reproducibility of such kits is problematic. Additionally, most false-negative patients are in the early stage of the disease. According to existing reports, the incubation period of COVID-19 is as long as 14 days, and in rare cases may be as long as 38 or 40 days (15, 16). Early clinical manifestations of COVID-19 are not typical. Once the disease enters the advanced stage, it progresses very rapidly. In the early stage of infection, when the number of viral copies are below, the detection threshold of qRT-PCR, false-negative results are inevitable.

### Many Specimens Are Not Kept at Optimal Conditions

According to China's regulations on the management of infectious diseases, specimens of positive cases must be sent to the Center for Disease Control and Prevention to confirm diagnosis; however, long-term transport of specimens is likely to cause degradation of viral RNA. As such, China eventually allowed these specimens to be tested by the testing department of any medical institution, as long as the testing department is equipped with a qualified clinical PCR laboratory and passes inspection for virus containment. However, prior to the outbreak of this pandemic, most clinical laboratories operated to meet clinical needs, with only a few having spare testing capacities. When the number of suspected cases reaches 100,000, and the number of daily tests exceeds the laboratory's testing capacity, testing has to be postponed on some specimens. It is difficult to send specimens for testing in a timely manner, and a significant number of specimens have to be delayed until the maximum allowable delay time prior to testing is reached. Additionally, some specimens are so delayed that the optimal time window for testing is missed. Presently, with the rapid expansion and renovation of laboratories, the process of specimen transport and testing has significantly improved.

### It Is Difficult to Control the Timing of Specimen Collection

Previous studies on the SARS coronavirus have shown that within 5 days prior to disease onset, the positive rate of viral NAT is high in specimen samples of the upper respiratory tract, which are collected using a nasopharyngeal aspirator or pharyngeal swab. As the disease progresses, the positive rate increases in stool specimen. A similar trend was observed for SARS-CoV-2 NAT; pharyngeal virus shedding was very high during the first week of symptoms, with a peak at 7.11 × 10^8^ RNA copies per throat swab on day 4. Infectious virus was readily isolated from samples derived from the throat or lung, but not from stool samples—in spite of high concentrations of virus RNA. Blood and urine samples never yielded the virus (17). Thus, a comprehensive understanding of the medical history may help us to control the sampling time.

### Selection of Specimen Collection Sites Still Needs to Be Improved

Wenling Wang detected the viral RNA of 1,070 different types of clinical specimens from 205 patients with COVID-19. Bronchoalveolar lavage fluid specimens showed the highest positive rates (14 of 15; 93%), followed by sputum (72 of 104; 72%), nasal swabs (5 of 8; 63%), fibrobronchoscope brush biopsy (6 of 13; 46%), pharyngeal swabs (126 of 398; 32%), feces (44 of 153; 29%), and blood (3 of 307; 1%). None of the 72 urine specimens tested positive (18). Kelvin Kai-Wang To also reported that salivary viral load was highest during the first week after symptom onset and subsequently declined with time (19). Most importantly, saliva samples are a non-invasive specimen more acceptable to patients and health-care workers; gathering nasopharyngeal and nasal swabs can cause discomfort for patients and put health-care workers at risk. Given that some patients do not have respiratory symptoms, such as a cough or expectoration, during the entire course of the disease, saliva samples could be a good choice for viral NAT. For suspected patients with gastrointestinal symptoms, stool or anal swabs can be used to collect specimens for viral NAT (20).

## Strategies for Improving the Diagnosis of COVID-19 (Figure 2)

### Combining Other Serological Indicators With NAT for a Comprehensive Evaluation

IgM and IgA are the first antibody isotypes detected 1 week following symptom onset, followed by IgG, which typically arise 2 weeks following symptom onset; thus, the serology test may only indicate past infection (19, 21, 22). IgM antibody was present in the body for 1 month or even longer and then gradually decreased until it was lower than the detection limit. IgG antibody is usually produced in about 10 days, but the time it will persist in the body remains unclear. However, after treatment, no significant difference in the level of IgM and IgG antibodies was found between nucleic acid-positive and negative patients (23). Due to cross-reactivity, the test of IgG/IgM always had many false positives, and moreover, the serology test's reliability issue associated with different brands of products is a real problem. Even though there was a study that showed that the total coincidence rate between antibody test and NAT in diagnosis of SARS-CoV-2 infection was 88.03% (24), we should combine both of them for a comprehensive evaluation. Additionally, other laboratory indicators such as inflammation and coagulation indicators are supportive diagnosis findings. Combined analyses of the levels of white blood cells, lymphocytes, C-reactionprotein (CRP), and serum amyloid protein A can improve the specificity and sensitivity of COVID-19 diagnosis (25–27). Moreover, it has a high reference value for the diagnosis of severe and critically ill patients with COVID-19. Inflammatory indicators such as lymphocyte subtypes and interleukin (IL)-6 can also be used to aid in diagnosis (28–30).

**Figure 2:**
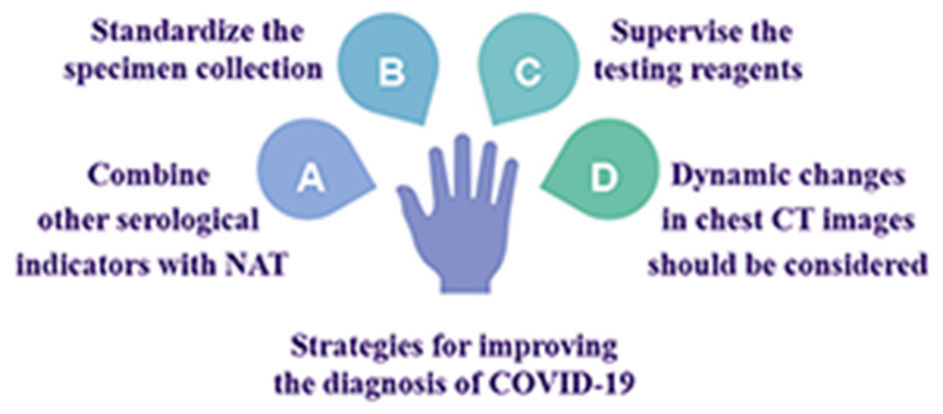
Strategies for improving the diagnosis of COVID-19.

### Specimen Collection Methods and Processes Should Be Standardized

In areas where conditions permit, specimen collection from other body sites, especially in patients with comorbid gastrointestinal symptoms, should be improved as much as possible through standardized collection and testing procedures of SARS-CoV-2 nucleic acid; this may prove to be beneficial by reducing false-negative results. When comparing clinical specimens from different body sites, further regulations on specimen sources is recommended in order to standardize clinical trials. It is necessary to standardize the specimen collection process through formal training and supervision so that specimen collection and preservation, prior to testing, draws more attention. We can follow the standards of the UK Biobank (http://www.ukbiobank.ac.uk/) to standardize our operating procedures, from which we can learn about the international standards and best practices.

### Testing Reagents Should Be Subjected to More Rigorous Supervision and Approval Procedures

For technology used in testing, no matter how scientifically sound the working principle, practical implementation is impossible without testing the equipment and reagents. Therefore, positive and negative controls, and inter-batch differences of kits, have significant importance on the quality of kits produced by different manufacturers. Proper positive, negative, and inhibition controls for extraction and amplification should be set in every test to ensure quality results (31, 32). After an exponential growth stage, the pandemic situation tends to become stable. Relevant governmental departments should require stringent supervision of various testing kits. Additionally, they should encourage each product testing laboratory, and clinical PCR laboratory, to cooperate with the clinical testing center in order to achieve inter-laboratory quality assessment resulting in systematic evaluations.

### Dynamic Changes in Chest CT Images Should Be Considered When Evaluating Disease Progression

With progression of the COVID-19 pandemic, atypical clinical cases are gradually increasing. NAT is prone to interference by various external factors that likely lead to inaccurate results, which may cause a certain degree of missed diagnoses of COVID-19. The dynamic changes of chest computed tomography (CT) images, combined with clinical manifestations, can be used as an effective complementary approach in confirming a diagnosis (33, 34). Therefore, in the *Novel Coronavirus Pneumonia Diagnosis and Treatment Plan* (5th trial version), clinical diagnosis criteria were added to the diagnostic criteria of Hubei Province, which, for the first time, also included CT imaging results. However, for infectious diseases, pathogen testing is the gold standard for confirming a diagnosis. The use of CT imaging alone, to distinguish COVID-19 from other viral pneumonia, lacks reliable criteria. In view of the actual circumstances and local conditions, this issue should be addressed in a comprehensive manner. Firstly, for patients who present with early and mild respiratory symptoms, but do not exhibit changes in chest CT scans, COVID-19 should not be ruled out in a subjective manner, without considering the importance of NAT. Secondly, in severely affected regions, the clinical diagnosis criteria for Hubei Province can be used as a standard reference, and more attention should be placed on CT scans (35). When patients exhibit a typical CT pattern of the lung fields, and the disease progresses rapidly, great precautions should be taken even when these patients test negative for SARS-CoV-2 nucleic acid (36). In such cases, patients should be immediately hospitalized, quarantined, and treated as per the *Novel Coronavirus Pneumonia Diagnosis and Treatment Plan*. Meanwhile, viral NAT should be conducted repeatedly with multi-site specimens, in order to avoid, as much as possible, a missed diagnosis. In summary, pathogen testing is an important approach in the detection of SARS-CoV-2; however, it should not be used alone to confirm diagnosis. Given that the virus is not yet well understood, attention should be more focused on the early clinical symptoms and imaging findings when an effective screening method for the incubation period is not available. To this end, NAT would serve as the gatekeeper to clinical diagnosis and should be combined with multiple laboratory tests to improve detection sensitivity.

## Factors Leading to False-Negative NAT Results and Considerations for Management of COVID-19

### Effective Collection of Specimen, Preservation of Viral Nucleic Acids, *in vitro* Diagnostic Reagents, and Clinical Laboratories

The following mistakes should be avoided: (1) *inappropriate collection sites*. When using oropharyngeal swabs for specimen collection, the collection depth is not enough. When nasopharyngeal swabs fail to reach the deep region of the nasal cavity, most of the collected cells may be virus-free cells (37); (2) *sampling swabs made of inappropriate materials*. Synthetic fibers such as polyethylene (PE), polyester, and polypropylene fibers are recommended materials for swab tips. However, natural fibers such as cotton (which strongly absorbs proteins that are not easy to elute) and nylon (which poorly absorbs water, resulting in insufficient specimen sampling) are used to make swab tips (38); (3) *virus preservation tubes are incorrectly used*. Polypropylene or polyethylene tubes, which are prone to adsorb nucleic acids (DNA/RNA), are mistakenly used to make tubes, resulting in reduced concentrations of nucleic acids. In practice, the use of polyethylene-propylene polymers, and some specially treated polypropylene plastic containers, is recommended in order to preserve viral nucleic acids. Errors in the above procedures may give rise to false-negative results. Additionally, reliable *in vitro* diagnostic reagents should be used. Some reagent manufacturers do not devote much time to developing reagents and do not use standard clinical specimens for necessary validation. As such, reagents may not be fully optimized causing large inter-batch differences in reagent quality. Lastly, standardized clinical laboratories are needed. The conditions of transporting and preserving specimens, standardized operations of clinical laboratories, interpretation of results, and quality control are also key factors that ensure accurate and reliable test results (39).

### The Immune System Is an Important Defense Mechanism Against Pathogenic Microorganisms in Humans

The strength of the immune system determines the severity and prognosis of a disease. Viruses are tiny non-cellular organisms that lack cellular structures, and as such, they require host cells to proliferate. The human body's immune system plays a pivotal role in killing viruses. COVID-19 patients suffer from immune disturbances, with the number of neutrophils and the levels of D-dimer, urea nitrogen, and creatinine continually increasing and the number of lymphocytes continually decreasing. Meanwhile, patients develop a cytokine storm due to the large production of the inflammatory factors IL-6, IL-10, and Granulocyte Macrophage Colony Stimulating Factor (GM-CSF). A cytokine storm is a severe systemic reaction caused by the over-activation of the immune system due to infection, drugs, or diseases, which can cause multiple organ failure and even death (40, 41). A cytokine storm occurs rapidly; that is why severe COVID-19 patients are prone to becoming critically ill in a very short period (42). Clinical trials have shown that the IL-6R antibody (tocilizumab) has a good therapeutic efficacy for COVID-19 (43). Therefore, when treating severe COVID-19, the immunomodulating properties of cytokines should be tested; based on the cytokine testing results, appropriate immunotherapies may be conducted in order to prevent the occurrence of cytokine storms, which is likely to alleviate disease severity in patients. After infecting the body, the virus enters the throat through the nasal and oral cavities, travels to the trachea and bronchi, and finally reaches the alveoli. Involvement of multiple immune mechanisms results in the development of different serious symptoms in infected individuals, while viral loads at various body sites vary with the change of disease severity, thereby leading to different durations of positive NAT results for SARS-CoV-2 (44).

### The Impact of Psychological Factors on the Rehabilitation of COVID-19 Patients Should Be Taken in Consideration

When patients develop psychological problems, their immunity declines, causing a corresponding decrease in the ability to kill the virus, resulting in a prolonged course of the disease and possible relapses and increased disease severity. Studies have shown that SARS-CoV-2 is highly threatening, has a long incubation period, and can place patients in a dangerous condition. To date, there is no exceptionally effective treatment for COVID-19. Confirmed COVID-19 patients, and quarantined suspected patients, suffer from different degrees of anxiety, nervousness, and desperation; this may be attributed to the fact that SARS-CoV-2 is an emerging novel virus with uncertainty and diversity, which can have a negative, and unhealthy, psychological impact on a patient's health. This suggests that it is necessary to pay attention to the mental health of both confirmed COVID-19 patients and quarantined suspected patients, through psychological evaluations and consultations. NAT testing for patients with psychological problems should be carried out when patients' mentality was relatively stable, and comprehensive judgment should be made in combination with other diagnostic testing.

## Recent Developments of NAT

Lab-based techniques still dominate the field of virus diagnostics. Nucleic acid amplification tests (NAATs), sequencing (including next-generation sequencing), and different antigen detection methods are now coming into the lab to complement classical methods (45). Due to high specificity, faster turnaround times, and absence of limitations posed by the need for susceptible cell lines, point-of-care (POC) PCR-based lateral flow assay and isothermal NAAT have played an important role in most clinical settings (46). Results can be produced in minutes by the POC molecular diagnostic tests, and therefore, it is convenient to operate for patients who have clinical symptoms and epidemiological risk factors for COVID-19. However, these tests can only be performed on specific instruments and amplify a single genomic target of SARS-CoV-2, result in less sensitivity and specificity as compared to traditional qRT-PCR based on molecular diagnostics. Novel biological sensors should also be developed as rapid, sensitive, and low-cost POC diagnostic devices for SARS-CoV-2 detection in the near future (47). The system consists of an immobilized biological component to recognize a target biomarker in the sample and a transducer to convert the corresponding biological signal into an electrical signal. Future biosensing devices for SARS-CoV-2 should also have limited sample processing steps and be able to deliver quick and accurate POC diagnoses.

Large-scale population screening using high-throughput RT-PCR for COVID-19 infection is generally considered a necessary part of an exit strategy from the coronavirus lockdown, such as testing Wuhan city residents and other cities for virus containment. Specimen pooling is a method of screening large number of patients for an infection and typically involves combining multiple patient specimens into a single test sample, then testing multiple such samples (48). Pooled specimen testing would enable substantial savings in reagent costs, technical burden, and time to generate laboratory results (49). Pooling swab specimens did not lower the sensitivity of PCR testing but actually increased the viral concentration when more than one positive sample was present in the same pool (50). However, serology tests were not suitable for this purpose because the concentrations of antibodies were diluted after pooling.

## Conclusion

In summary, during the clinical diagnosis of COVID-19, it is necessary to carefully analyze patients' epidemiological history, clinical manifestations, and dynamic changes in the results of auxiliary examinations in order to conduct comprehensive evaluations. For highly suspected cases, reliance on NAT results of upper respiratory tract specimens as the sole diagnostic standard to confirm a diagnosis should be avoided, as it can lead to missed diagnoses. This could lead to haste lifting of the quarantine, which in turn would result in further spread of the pandemic. In the meantime, after COVID-19 patients are discharged from the hospital, they should be placed on an additional 14-day quarantine, while monitoring their health condition. Comprehensive analyses of serological and imaging findings should be performed to guide the formulation of an accurate clinical diagnosis, treatment plan, and monitoring therapeutic efficacy, in an attempt to achieve early diagnosis, containment, and treatment of the disease, thereby effectively reducing progression of the pandemic.

## Author Contributions

HW designed the study and prepared the manuscript. GL and JZ reviewed the manuscript. All authors contributed to the article and approved the submitted version.

## Conflict of Interest

The authors declare that the research was conducted in the absence of any commercial or financial relationships that could be construed as a potential conflict of interest.
